# Features of the Structure and Electrophysical Properties of Solid Solutions of the System (1-x-y) NaNbO_3_-xKNbO_3_-yCd_0.5_NbO_3_

**DOI:** 10.3390/ma14144009

**Published:** 2021-07-17

**Authors:** Konstantin Andryushin, Lidiya Shilkina, Inna Andryushina, Alexandr Nagaenko, Maxim Moysa, Svetlana Dudkina, Larisa Reznichenko

**Affiliations:** 1 Research Institute of Physics, Southern Federal University, 344090 Rostov-on-Don, Russia; lid-shilkina@yandex.ru (L.S.); futur6@mail.ru (I.A.); moysa@sfedu.ru (M.M.); s.i.dudkina@yandex.ru (S.D.); lareznichenko@sfedu.ru (L.R.); 2 Institute of High Technology and Piezo Technic, Southern Federal University, 344090 Rostov-on-Don, Russia; nagaenko@sfedu.ru

**Keywords:** ferroelectric materials, KNN, cadmium niobate, structure, microstructure, electrophysical properties, dielectric spectroscopy

## Abstract

Ferroelectric ceramic materials based on the (1-x-y) NaNbO_3_-xKNbO_3_-yCd_0.5_NbO_3_ system (*x* = 0.05–0.65, *y* = 0.025–0.30, Δ*x* = 0.05) were obtained by a two-stage solid-phase synthesis followed by sintering using conventional ceramic technology. It was found that the region of pure solid solutions extends to *x* = 0.70 at *y* = 0.05 and, with increasing *y*, it narrows down to *x* ≤ 0.10 at *y* = 0.25. Going out beyond the specified concentrations leads to the formation of a heterogeneous region. It is shown that the grain landscape of all studied ceramics is formed during recrystallization sintering in the presence of a liquid phase, the source of which is unreacted components (Na_2_CO_3_ with T_melt._ = 1126 K, K_2_CO_3_ with T_melt._ = 1164 K, KOH with T_melt._ = 677 K) and low-melting eutectics in niobate mixtures (NaNbO_3_, T_melt_. = 1260 K, KNbO_3_, T_melt._ = 1118 K). A study of the electrophysical properties at room temperature showed the nonmonotonic behavior of all dependences with extrema near symmetry transitions, which corresponds to the logic of changes in the electrophysical parameters in systems with morphotropic phase boundaries. An analysis of the evolution of dielectric spectra made it possible to distinguish three groups of solid solutions: classical ferroelectrics (*y* = 0.05–0.10), ferroelectrics with a diffuse phase transition (*y* = 0.30), and ferroelectrics relaxors (*y* = 0.15–0.25). A conclusion about the expediency of using the obtained data in the development of materials and devices based on such materials has been made.

## 1. Introduction

The rapid development of piezoelectric instrumentation in the past 50 years has led to the widespread use of ceramics based on the PZT system (PbZr_1-x_Ti_x_O_3_) in sensors, actuators, electronic and microelectronic devices. This is due to the high values of the piezoelectric constants and the Curie temperature [1,2,3,4,5]. It should be noted that, due to the high toxicity of lead, which is practically not excreted from living organisms, in recent decades, an active search for alternative materials has been carried out [6,7,8,9,10,11]. Compositions based on sodium–potassium niobates (KNN) ((K, Na) NbO_3_) [12,13,14,15,16], which have a number of unique properties: low dielectric constant (100–300), high speed of sound (*V_R_* ≈ 6 km/s) with a wide range of mechanical quality factors (from units to thousands) and low specific weight (<4.5 g/cm^3^). However, these lead-free compositions occupy only a narrow segment of piezoelectric applications, such as microwave technology, and there are also a number of disadvantages to these materials becoming commercially disattractive. The indicated negative aspects of this object include its weak compaction at a low sintering temperature and the volatility of Na and K ions [17], which facilitates the formation of structural defects and, as a consequence, a decrease in the expected electrophysical characteristics of ceramic objects. In order to eliminate these drawbacks, as well as expand the existing spectrum of properties of the binary system (K, Na)NbO_3_, it seems relevant to design a three-component system and to establish correlations between the compositional, structural, and electrophysical properties of the obtained solid solutions (SS). However, it should be noted that the complication of the (K, Na) NbO_3_ binary system by the introduction of Cd_0.5_NbO_3_ does not eliminate its disadvantages, but leads to an increase in the dimension of the geometric image, which determines a large number of possible compositions. The latter makes it possible to increase the dimensionality of the morphotropic region and, as a consequence, the number of media with potentially demanded properties. This is similar to how this process occurs, for example, in the SS based on PZT [18,19] and other [20,21,22], etc. Additionally, with the introduction of cadmium niobate up to 30 mol. %, one can expect the absence of a reconstructive transition from the perovskite to the columbite structural type to which it belongs and the specificity of the properties inherent in this family can have a positive effect on the properties of the final three-component SS.

## 2. Materials and Methods

### 2.1. Fabrication of Samples

The objects of study were SS based on the system (1-x-y) NaNbO_3_-xKNbO_3_-yCdNb_2_O_6_, in which the first two components are NaNbO_3_ (space group *Pbma*, *a* = 5.5687 Å, *b* = 15.523 Å, *c* = 5.5047 Å) [23] and KNbO_3_ (space group *Cm2m*, *a* = 5.695 Å, *b* = 5.7213 Å, *c* = 3.9739 Å) [24] crystallize in the perovskite structural type with an orthorhombic distorted perovskite subcell. The third component, CdNb_2_O_6_ (*y*), belongs to the structural type of columbite where crystal lattice symmetry is rhombic (space group *Pcan*). Cell parameters: *a* = 5.848 Å, *b* = 14.7817 Å, *c* = 5.1419 Å [25].

Further, when writing the chemical formula of this compound, we reduce it to the structural type of perovskite, ABO_3_ (Cd_0.5_NbO_3_). Reagent-grade sodium hydrocarbonate (NaHCO_3_, 99%), potassium hydrocarbonate (KHCO_3_, 99%), niobium (Nb_2_O_5_, 99%) and cadmium (CdO, 99%) oxides were used as raw materials.

**Figure 1:**
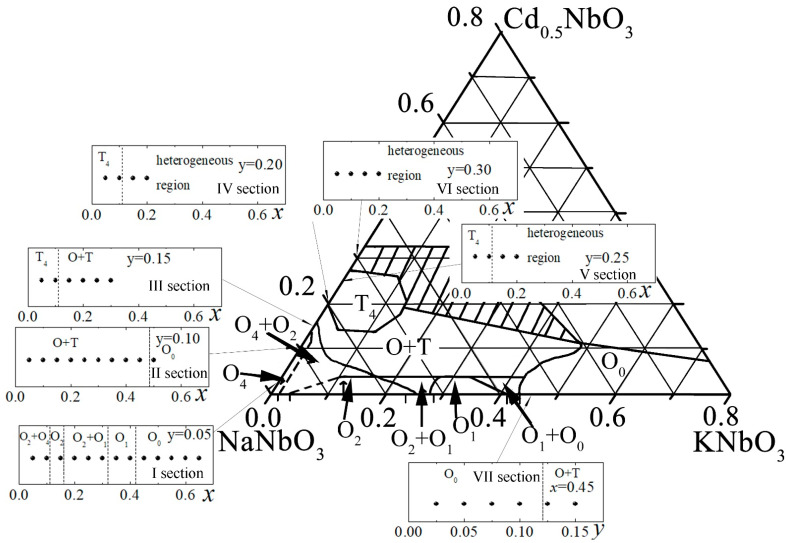
Phase diagram of the triple three-component system (1-x-y) NaNbO_3_-xKNbO_3_-yCd_0.5_NbO_3_.

Figure 1 shows the Gibbs triangle with selected sections (I-VII), experimental points, as well as highlighted boundaries of morphotropic regions (phases: T—tetragonal, O—orthorhombic, heterogeneous (with impurities) region—shaded): I section with *y* = 0.05, *x* = 0.05–0.65, Δ*x* = 0.05; II section with *y* = 0.10, *x* = 0.05–0.50, Δ*x* = 0.05; III section with *y* = 0.15, *x* = 0.05–0.30, Δ*x* = 0.05; IV section with *y* = 0.20, *x* = 0.05–0.20, Δ*x* = 0.05; V section with *y* = 0.25, *x* = 0.05–0.20, Δ*x* = 0.05; VI section with y = 0.30, *x* = 0.05–0.20, Δ*x* = 0.05; VII section with *y* = 0.025–0.150, *x* = 0.45.

We have considered the part of the Gibbs diagram adjacent to the NaNbO_3_ vertex. The choice of this fragment of the phase picture of the system was determined by the search for materials with characteristics similar to those realized in SS based on sodium–potassium and sodium–cadmium niobates [1,26,27].

The objects were obtained by double solid-phase synthesis with firing temperatures T_synt. 1_ = 1220 K, τ = 5 h, T_synt. 2_ = 1240 K, τ = 10 h and by sintering using conventional ceramic technology at T_sint._ = (1400–1510) K, depending on the composition and in accordance with the recommendations [28]. The search-measuring samples were made in the form of the disks (Ø10·1 or Ø10·0.5 mm) with the silver-containing electrodes. Metallization was carried out by double firing a silver-containing paste at a temperature of 1070 K for 0.5 h.

The samples were polarized in a polyethylene siloxane liquid at the temperature (390–410) K and the electric field strength (3–4) kV/mm for 15 min, followed by cooling under the field to (363–353) K for (25–30) min.

### 2.2. Methods of Studying Samples

The X-ray diffraction studies were performed by the powder diffraction on a DRON-3 diffractometer (JSC "Innovation center "Burevestnik", St. Petersburg, Russia) using the *Co_K_*_α_ radiation (geometry of Bragg-Brentano). Crushed ceramic objects were investigated, which made it possible to exclude the influence of surface effects, stresses and textures arising in the process of obtaining ceramics.

The X-ray density was calculated by the formula: *ρ*_x-ray_ = 1.66 × *M*/*V*, where *M* is the molecular weight per cell and *V* is the volume of the cell. The relative density was determined by the formula: *ρ*_rel._ = (*ρ*_exp._/*ρ*_x-ray_)·100%.

A JSM-6390L scanning electron microscope (JEOL Ltd., Tokyo, Japan) with a system of the microanalyzers from the Oxford Instruments (Great Britain, UK) was used to study the microstructure of the sample chips. The microscope resolution was up to 1.2 nm at an accelerating voltage of 30 kV (the image in the secondary electrons), the accelerating voltage range was from 0.5 to 30 kV, the magnification from ×10 to ×1,000,000 and the beam current was up to 200 nA.

The temperature dependences of the real and imaginary parts of the relative complex permittivity ε*/*ε*_0_ = *ε*′/*ε*_0_ − *iε*″/*ε*_0_ (*ε*_0_ = 8.75 × 10^−12^ F/m—the dielectric constant) of the samples at T = (300–800) K and the frequency range *f* = (25–10^6^) Hz, were obtained using a measuring stand based on an Agilent 4980A LCR meter.

The electrophysical parameters of the SS at T = (300–943) K were measured using an Agilent E4980A precision LCR meter (Keysight Technologies, Malaysia) by the resonance–antiresonance method [29]. The relative permittivity of the polarized (*ε*_33_*^T^/ε*_0_) samples, the dielectric losses in a weak field (the tangent of dielectric loss angle, tg*δ*), the piezomodule (|*d*_31_|), the piezoelectric coefficient (the piezoelectric sensitivity) (|*g*_31_|), the electromechanical coupling coefficient of the planar vibration modes (*K_p_*), the mechanical quality factor (*Q_M_*), the Young’s modulus (*Y*_11_*^E^*) and the speed of sound (V_1_^E^) were determined. The piezomodule *d*_33_ and, respectively, the piezoelectric coefficient *g*_33_ were measured at room temperature by the quasistatic method using Piezo *d*_33_ Test System (YE2730A d_33_ METER). The measurement errors of the electrophysical parameters have the following values: *ε*_33_*^T^/ε*_0_ ≤ ±1.5%, *K_p_* ≤ ±2.0%, |*d*_31_| ≤ ±4.0%, *Q_M_* ≤ ±12%; *Y*_11_*^E^* ≤ ±0.7%.

## 3. Results and Discussion

### 3.1. Crystal Structure

It has been established that up to *y* = 0.25, the relative density of ceramics is ~90%. Samples from various fragments of the samples showed a reproduction of their integral density and there was no scatter in the density values between the samples (disks) cut from the middle parts of the pillars. All this indicates a high homogeneity of the studied samples and the reliability of the results obtained in the work. X-ray phase analysis showed that the region of pure SS extends to *x* = 0.70 at *y* = 0.05 and with increasing *y* it narrows down to *x* ≤ 0.10 at *y* = 0.25. Going out beyond the indicated concentrations leads to the formation of a heterogeneous region (it is shaded in the Gibbs triangle (Figure 1)). The latter includes, along with SS with a perovskite structure, Cd_0.5_NbO_3_, the content of which increases with increasing *x* at *y* = 0.25 and decreases at *y* = 0.30.

**Figure 2:**
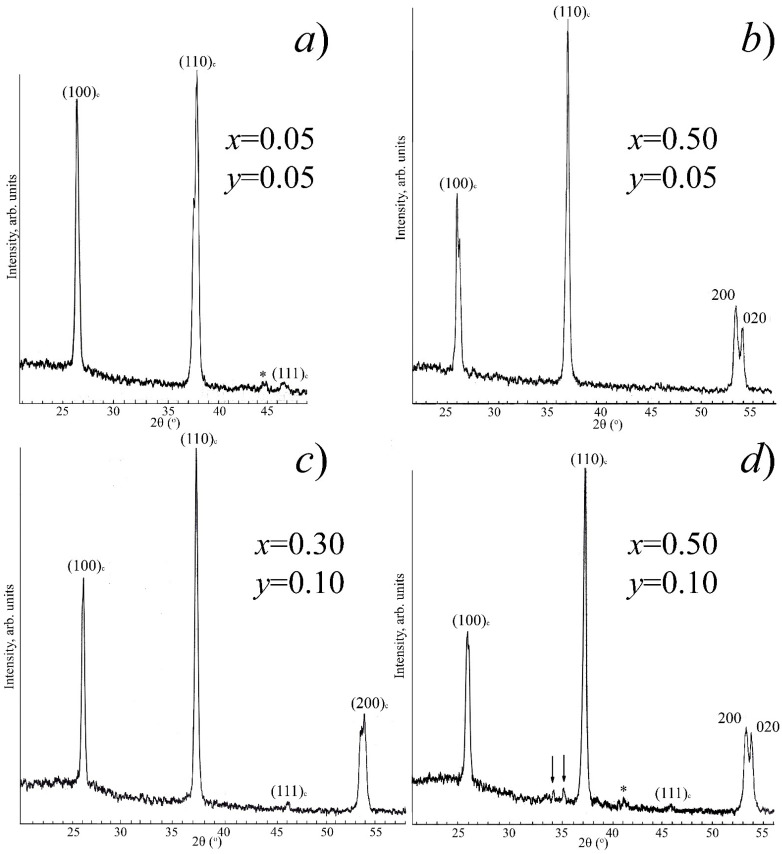
Fragments of X-ray diffraction patterns of SS of I (**a**,**b**), II (**c**,**d**) sections.

**Figure 3:**
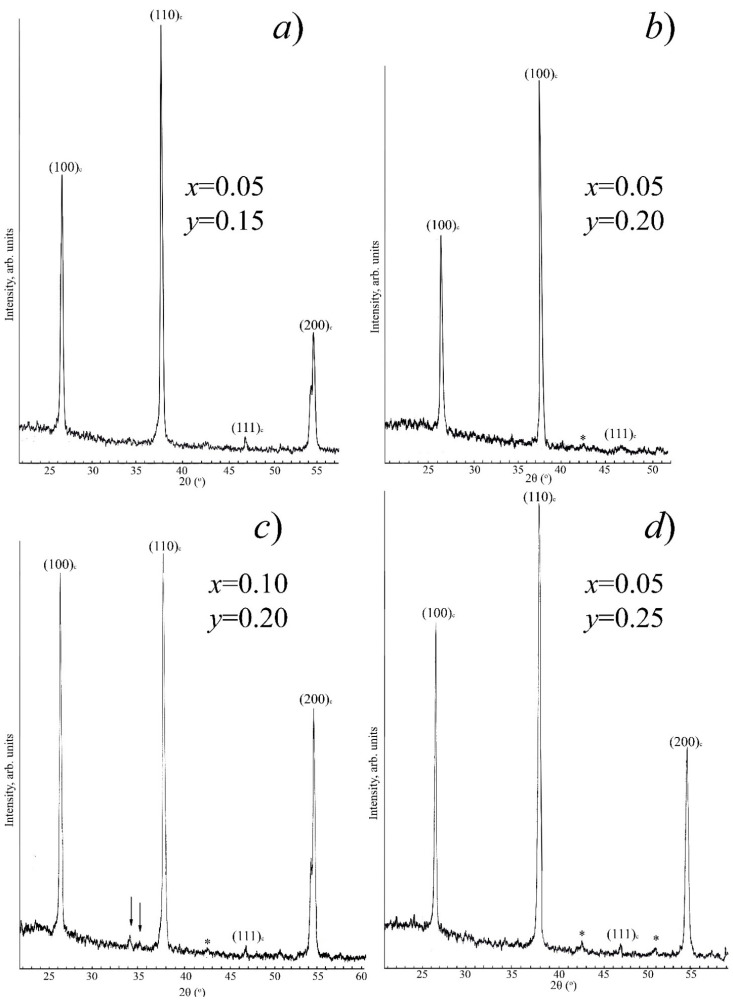
Fragments of X-ray diffraction patterns of III (**a**), IV (**b**,**c**) and V (**d**) sections.

The most intense X-ray peaks of impurity (extraneous) phases that are formed in the system under study are located in the range 2θ = 21–49 (^o^). In Figure 2 and Figure 3, for illustration, fragments of X-ray diffraction patterns in this angular range of SS from various sections of the ternary system are presented: Figure 2a-I section (*x* = 0.05, *y* = 0.05); Figure 2b-I section (*x* = 0.50, *y* = 0.05); Figure 2c,d-II section (*x* = 0.30, *y* = 0.10), (*x* = 0.50, *y* = 0.10); Figure 3a-III section (*x* = 0.05, *y* = 0.15); Figure 3b,c-IV section (*x* = 0.05, *y* = 0.20), (*x* = 0.10, *y* = 0.20); and Figure 3d-V section (*x* = 0.05, *y* = 0.25). Asterisks indicate superstructure peaks; arrows indicate X-ray peaks of the K_2_Nb_8_O_21_ compound.

In the region of pure SS, it was possible to distinguish 6 regions of different phase composition: 1—orthorhombic (O_4_) (the subscript shows the multiplication of the *b* axis of the monoclinic perovskite subcell) phase with perovskite cell parameters close to those of NaNbO_3_; 2—SS in which two O phases, O_4_ and O_2_, coexist with similar cell parameters, differing in the multiplication of the perovskite axis *b*; 3—O_2_ phase, in which only doubling of the *b* axis is observed; 4—O_1_—orthorhombic phase, in which the parameter *b* of the monoclinic perovskite subcell and the parameter *B* of the orthorhombic cell are equal, in the same way as in the quasi-binary system (1-x) NaNbO_3_-xKNbO_3_ at *x* ≥ 0.30 [27]; 5—SS in which O and tetragonal (T) phases coexist (cell multiplication in each of the phases could not be determined) and the cell parameters of the O phase change with increasing x in the same way as in the system (1-x) NaNbO_3_-xKNbO_3_ [27]; 6—T phase. Thus, it was found that when Cd_0.5_NbO_3_ is introduced at a concentration no more than 5 mol %, the phase picture is similar to the observed quasi-binary system KNN and, at *y* > 0.05, an additional T-phase appears.

### 3.2. Microstructure

**Figure 4:**
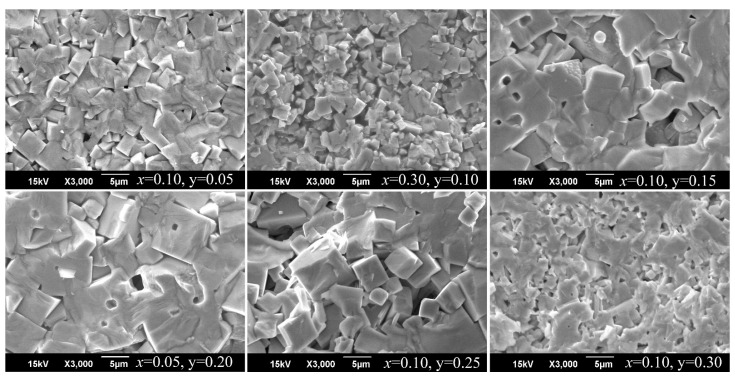
Fragments of the microstructure of various SS ceramics of the system (1-x-y) NaNbO_3_-xKNbO_3_-yCd_0.5_NbO_3_.

**Figure A1:**
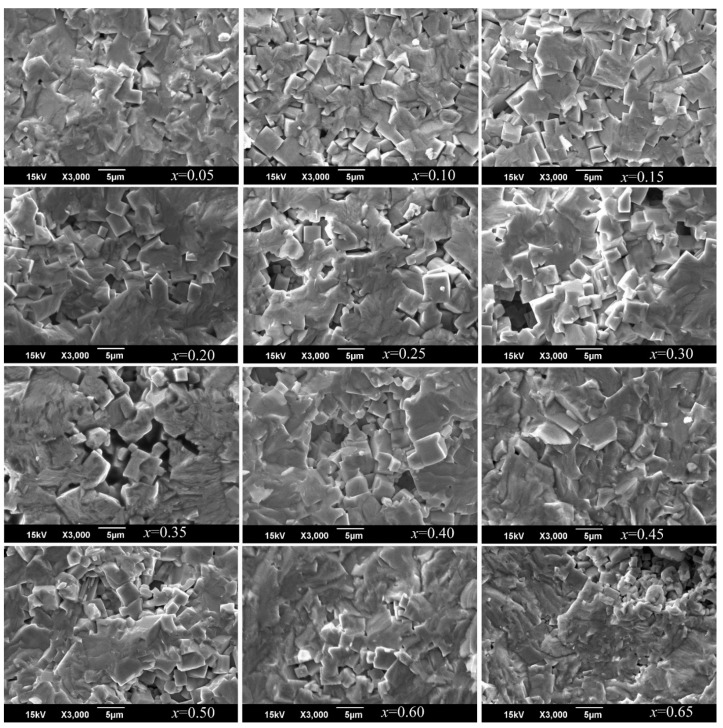
Fragments of the microstructure of SS ceramics of the system: (1-x-y) NaNbO_3_-xKNbO_3_-yCd_0.5_NbO_3_ at *y* = 0.05.

**Figure A2:**
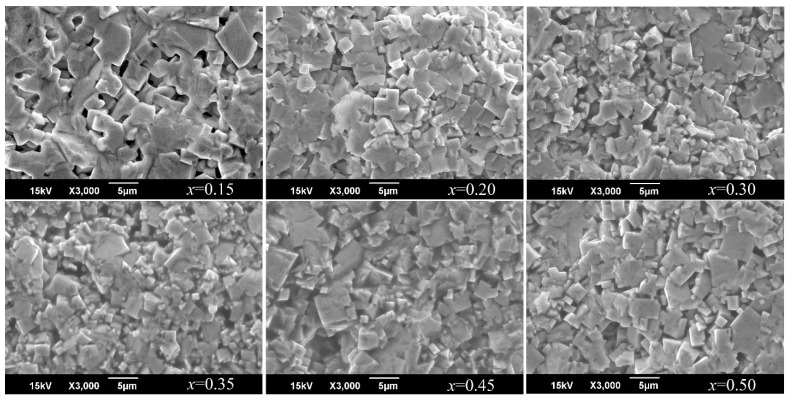
Fragments of the microstructure of SS ceramics of the system: (1-x-y) NaNbO_3_-xKNbO_3_-yCd_0.5_NbO_3_ at *y* = 0.10.

**Figure A3:**
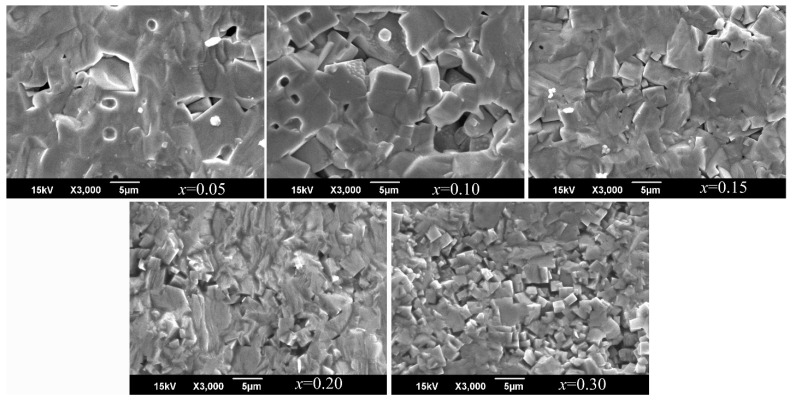
Fragments of the microstructure of SS ceramics of the system: (1-x-y) NaNbO_3_-xKNbO_3_-yCd_0.5_NbO_3_ at *y* = 0.15.

**Figure A4:**
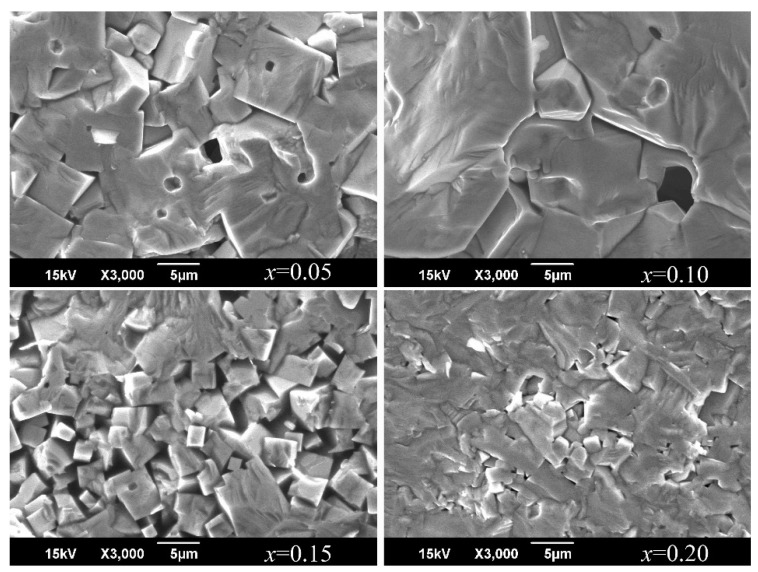
Fragments of the microstructure of SS ceramics of the system: (1-x-y) NaNbO_3_-xKNbO_3_-yCd_0.5_NbO_3_ at *y* = 0.20.

**Figure A5:**
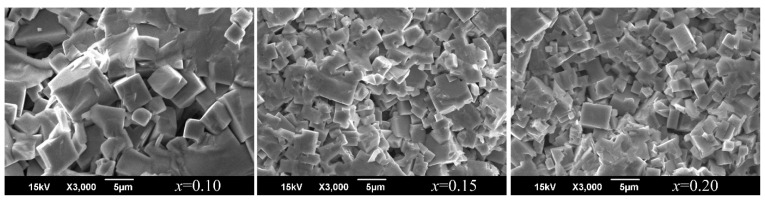
Fragments of the microstructure of SS ceramics of the (1-x-y) NaNbO_3_-xKNbO_3_-yCd_0.5_NbO_3_ at *y* = 0.25.

**Figure A6:**
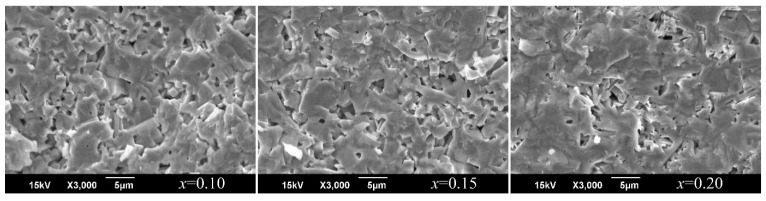
Fragments of the microstructure of SS ceramics of the system: (1-x-y) NaNbO_3_-xKNbO_3_-yCd_0.5_NbO_3_ at *y* = 0.30.

**Figure A7:**
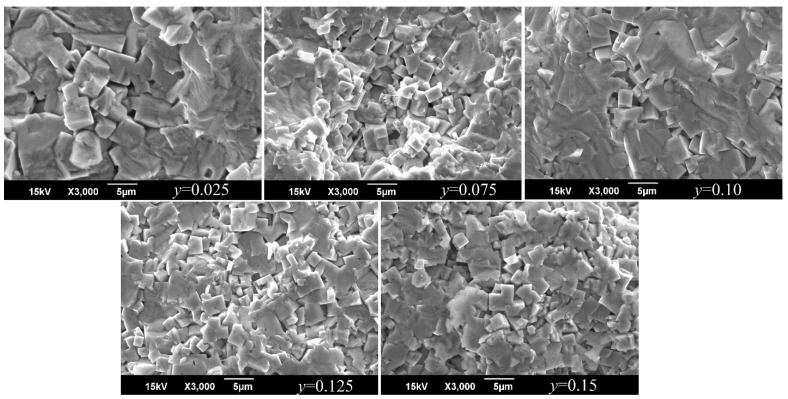
Fragments of the microstructure of SS ceramics of the system: (1-x-y) NaNbO_3_-xKNbO_3_-yCd_0.5_NbO_3_ at *x* = 0.45.

Figure 4 illustrates photographs of fragments of microstructures of some studied ceramics, the composition of which corresponds to SS of I–VI sections (for all studied SS—photos are presented in Appendix A. Figure A1, Figure A2, Figure A3, Figure A4, Figure A5, Figure A6 and Figure A7). In all cases, to one degree or another, an inhomogeneous structure of the grain field is noted with the formation of large crystallites in the form of “plates” and smaller ones, close in shape to cubic ones. The packing of grains is chaotic and mostly dense which provides a sufficiently high density of the ceramic framework. In some cases, loose structures with crystallites that are not interconnected by intercrystalline interlayers predominate.

The fact that in the process of recrystallization sintering, in all cases under consideration, grains of almost regular geometric shapes with clear edges are formed, indicates that the grains grow in the groundmass for which, in addition to solid grains and gaseous pores, there is a liquid phase (LP) [30,31] in which the grains are immersed. Most often, such ideomorphic grains are characteristic of secondary discontinuous recrystallization processes. The impurities located at the grain boundary at the recrystallization temperature form an active LP that interacts with the base material, playing the role of not so much a “lubricant” as a transport medium, that is, a solvent. The selective growth of large grains in this case occurs due to the usual dissolution and precipitation from solutions and not due to the movement of boundaries. At the same time, the liquid covering the surface of such grains with a film makes them acquire a certain growth shape and a regular facet identical to the habit of crystals growing from a molten solution. As a result, ideomorphic grains crystallize in the form of polyhedrons with practically rectilinear boundaries [32].

The formation of LP 2 is associated with the presence of unreacted components (Na_2_CO_3_ with T_melt._ = 1126 K, K_2_CO_3_ with T_melt._ = 1164 K, KOH with T_melt._ = 677 K) and low-melting eutectics in niobate charges (NaNbO_3_, T_melt._ = 1260 K, KNbO_3_, T_melt._ = 1118 K).

**Figure 5:**
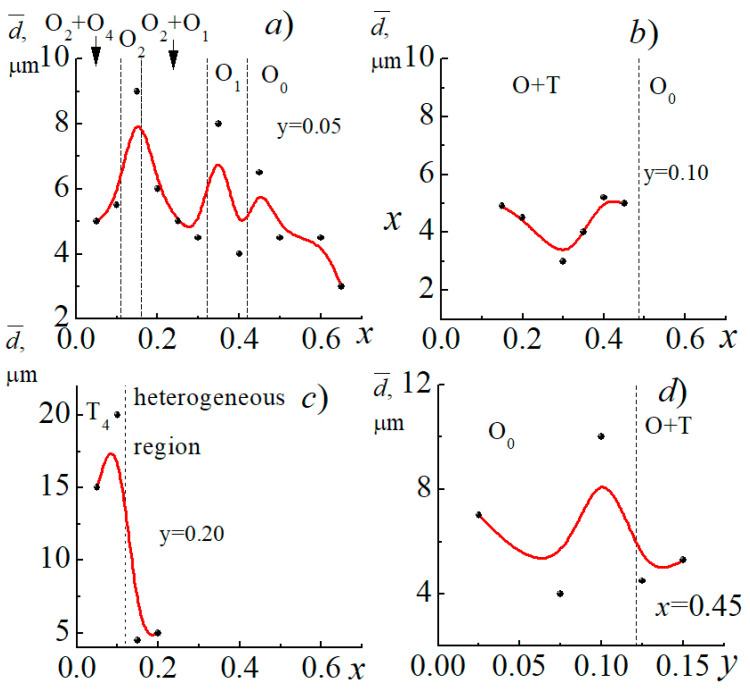
Dependences of the average size of large grains on the concentration of xKNbO_3_ (**a**–**c**) and Cd_0.5_NbO_3_ (**d**) (experimental points are marked with markers; red solid lines serve as guide to the eye).

As can be seen from Figure 4, the size and shape of crystallites and pores, the degree of bimodality of the grain landscape and other characteristics of microstructures depend on the qualitative and quantitative composition of the studied compositions, as well as the position of the SS on the phase diagrams of the corresponding sections of the analyzed three-component system. Thus, typical for the SS of the I section of the system is the extreme nature of the change in the average size, $\bar{d}$, of larger grains with maxima in the vicinity of *x* ~ 0.15 and *x* ~ 0.45, corresponding to phase transformations in this region of component concentration (Figure 5). A similar “behavior” of crystallites with increasing size in the vicinity of various structural instabilities is observed in other sections of the system. This demonstrates to the manifestation of the well-known correlation relationship “composition-structure-properties”. The observed can be explained by the greater mobility of structural elements of SS localized in morphotropic regions due to a decrease in spontaneous deformation here and, as a consequence, internal stresses in ferroelectric ceramics, which restrain grain growth [4].

### 3.3. Electrophysical Properties

**Figure 6:**
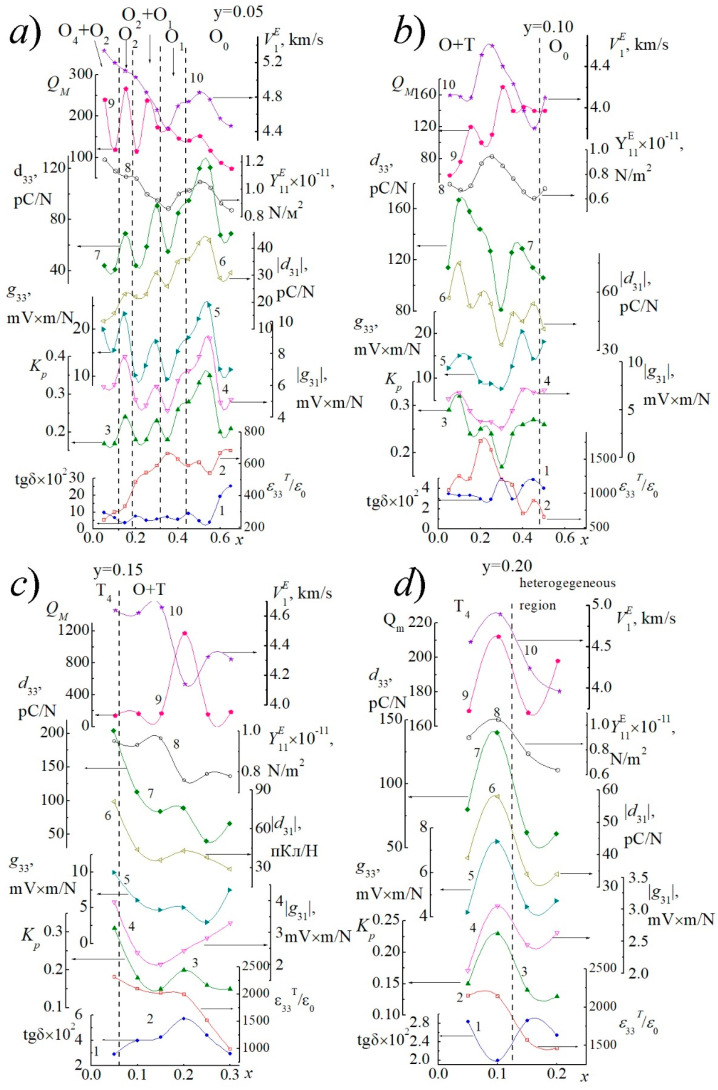
Dependences of the electrophysical parameters of piezoceramic materials of the studied SS on the concentration of KNbO_3_ (1—tg*δ*, 2—*ε*_33_*^T^*/*ε*_0_, 3—*K_p_*, 4—*g*_33_, 6—*d*_33_, 8—*Y*_11_*^E^*, 9—*Q_M_*, 10—*V_r_*) (**a**) *y* = 0.05; (**b**) *y* = 0.10; (**c**) *y* = 0.15; (**d**) *y* = 0.20 (experimental points are marked with markers. Solid lines serve as guide to the eye).

**Figure 7:**
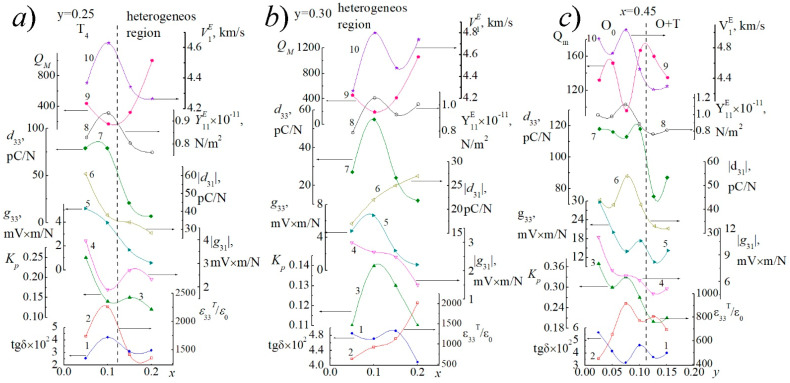
Dependences of the electrophysical parameters of piezoceramic materials of the studied SS on the concentration of KNbO_3_ (**a**—*y* = 0.25, **b**—*y* = 0.30) and Cd_0.5_NbO_3_ (**c**—*x* = 0.45). (1—tg*δ*, 2—*ε*_33_*^T^*/*ε*_0_, 3—*K_p_*, 4—*g*_33_, 6—*d*_33_, 8—*Y*_11_*^E^*, 9—*Q_M_*, 10—*V_r_*) (experimental points are marked with markers. Solid lines serve as guide to the eye).

Figure 6 and Figure 7 shows the dependences of the dielectric, piezoelectric, and ferroelastic characteristics of the studied SS on the concentration of KNbO_3_ (Figure 6a–d and Figure 7a,b) and Cd_0.5_NbO_3_ (Figure 7c). 

The nonmonotonic behavior of all dependences with extrema near symmetry transitions corresponds to the logic of changes in the electrophysical parameters in the systems with morphotropic phase boundaries. The irregularity of all dependencies is undoubtedly a consequence of the extreme complexity of the phase diagram of the system (Figure 1), with a large number of sequential phase transformations of different characters.

**Figure 8:**
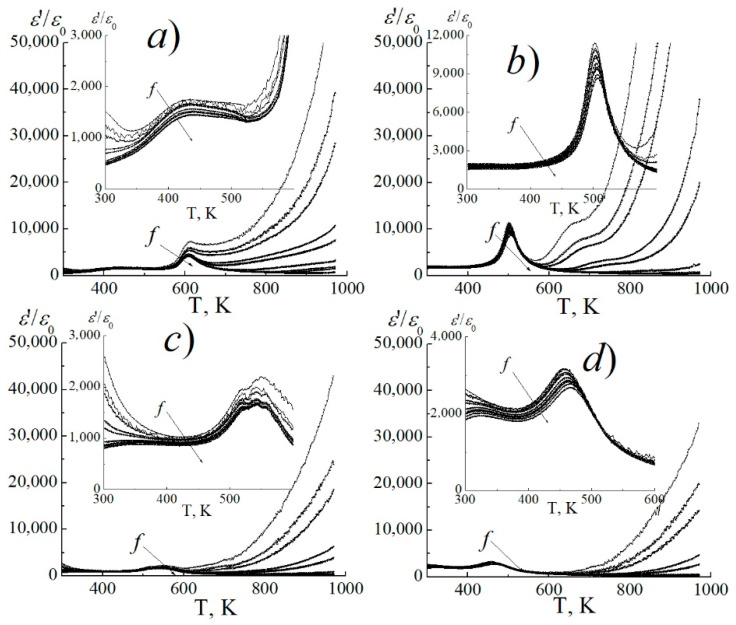
The most typical dependences of *ε*′/*ε*_0_ on the temperature in the frequency range *f* = (25–10^6^) Hz for three groups of SS (cooling mode) (**a**) *y* = 0.05, *x* = 0.15; (**b**) *y* = 0.15, *x* = 0.05; (**c**) *y* = 0.30, *x* = 0.20; (**d**) *y* = 0.15, *x* = 0.15.

An analysis of the most typical dielectric spectra (Figure 8) showed that, by the nature of the dependences *ε*′/*ε*_0_(T) at different *f*, three groups of SS can be distinguished: classical ferroelectrics with *y* = 0.05, 010; ferroelectric relaxors with *y* = 0.15–0.25; and ferroelectrics with a diffuse phase transition (PT) with *y* = 0.30 (Figure 8c, respectively).

In the first group, the *ε*′/*ε*_0_(T) dependences (Figure 8a) show the formation at the Curie temperature (T_c_) of a practically non-blurring and unchanging maximum *ε*′/*ε*_0_, which is most distinct at high *f*, characterized by the absence of dispersion *ε*′/*ε*_0_ to the left of T_c_, its appearance at the time of the PT and to the right of T_c_. Above T_c_, a rapid increase in *ε*′/*ε*_0_ is observed, starting from temperatures at the higher *f*. In addition to the main one, the presence of one more low-temperature maximum which is strongly diffuse, a dispersive maximum *ε*′/*ε*_0_ that does not change its position and is localized in the interval T = (350–550) K, depending on the composition, is noted.

The second group of SS is characterized by the formation of only one maximum *ε*′/*ε*_0_ at T_c_, which has a relaxation character (there is no low-temperature maximum) (Figure 8b) and is characterized by a shift in *ε*′/*ε*_0_ towards higher temperatures with increasing *f*. In this case, a weak dispersion *ε*′/*ε*_0_ is noticeable to the left of T_c_, which increases at T_c_ and disappears to the right of it, up to T ~ 550 K. In the Arrhenius coordinates, the dependence T_m_ (*f*) (where T_m_ is the temperature of this maximum *ε*′/*ε*_0_) is not linear. The last one indicates a non-Debye nature of relaxation, typical for ferroelectric relaxors. At the same time, this dependence is well described by the Vogel-Fulcher law: *f* = *f*_0_ exp[−*E_a_*/*k*(T_m_ − T_0_)], where *f*_0_ is the frequency of attempts to overcome the potential barrier, *E_a_* is the activation energy of the process, *k* is the Boltzmann constant, T_m_ is the maximum temperature and T_0_ is the Vogel-Fulcher temperature, interpreted as the temperature of “static freezing” of electric dipoles or transition to the state of a dipole glass. In our case, the following values were obtained: for a SS with *y* = 0.15: T_0_ ≈ (413–495) K, *f*_0_ = (10^9^–10^11^) Hz, *E_a_* = (0.013–0.04) eV; for SS with *y* = 0.20: T_0_ ≈ (424–467) K, *f*_0_ = (10^7^–10^11^) Hz, *E_a_* = (0.009–0.014) eV; and for a SS with *y* = 0.25: T_0_ ≈ (405–451) K, *f*_0_ = (10^8^–10^11^) Hz, *E_a_* = (0.003–0.009) eV.

## 4. Conclusions

Experimental solid-state samples based on the (1-x-y) NaNbO_3_-xKNbO_3_-yCdNb_2_O_6_ system were prepared by two-stage solid-phase synthesis followed by sintering using conventional ceramic technology. It was established by X-ray diffraction that the region of pure solid solutions extends to *x* = 0.70 at *y* = 0.05 and narrows down to *x* ≤ 0.10 with an increase in the Cd_0.5_NbO_3_ content to 25 mol %. Going out beyond the specified concentrations leads to the formation of a heterogeneous region. It is shown that the grain landscape of all studied ceramics is formed during recrystallization sintering in the presence of a liquid phase, the source of which is unreacted components (Na_2_CO_3_ with T_melt._ = 1126 K, K_2_CO_3_ with T_melt._ = 1164 K, KOH with T_melt._ = 677 K) and low-melting eutectics in niobate mixtures (NaNbO_3_, T_melt._ = 1260 K, KNbO_3_, T_melt._ = 1118 K).

When studying the electrophysical properties at room temperature, it was found that all dependences demonstrate nonmonotonic behavior with extrema near symmetry transitions. This corresponds to the logic of changes in the electrophysical parameters in systems with morphotropic phase boundaries. The analysis of dielectric spectra evolution made it possible to distinguish three groups of solid solutions: classical ferroelectrics (*y* = 0.05–0.10), ferroelectrics with a diffuse phase transition (*y* = 0.30) and ferroelectrics relaxors (*y* = 0.15–0.25) with the parameters of the Vogel-Fulcher law: T_0_ ≈ (413–495) K, *f*_0_ = (10^9^–10^11^) Hz, *E_a_* = (0.013–0.04) eV (*y* = 0.15); T_0_ ≈ (424–467) K, *f*_0_ = (10^7^–10^11^) Hz, *E_a_* = (0.009–0.014) eV (*y* = 0.20); T_0_ ≈ (405–451) K, *f*_0_ = (10^8^–10^11^) Hz, *E_a_* = (0.003–0.009) eV (*y* = 0.25).

The results obtained in this work should be taken into account when developing materials and devices based on such materials.
